# Resampling-Based Approaches to Study Variation in Morphological Modularity

**DOI:** 10.1371/journal.pone.0069376

**Published:** 2013-07-16

**Authors:** Carmelo Fruciano, Paolo Franchini, Axel Meyer

**Affiliations:** Department of Biology, University of Konstanz, Konstanz, Germany; BiK-F Biodiversity and Climate Research Center, Germany

## Abstract

Modularity has been suggested to be connected to evolvability because a higher degree of independence among parts allows them to evolve as separate units. Recently, the Escoufier RV coefficient has been proposed as a measure of the degree of integration between modules in multivariate morphometric datasets. However, it has been shown, using randomly simulated datasets, that the value of the RV coefficient depends on sample size. Also, so far there is no statistical test for the difference in the RV coefficient between *a priori* defined groups of observations. Here, we (1), using a rarefaction analysis, show that the value of the RV coefficient depends on sample size also in real geometric morphometric datasets; (2) propose a permutation procedure to test for the difference in the RV coefficient between *a priori* defined groups of observations; (3) show, through simulations, that such a permutation procedure has an appropriate Type I error; (4) suggest that a rarefaction procedure could be used to obtain sample-size-corrected values of the RV coefficient; and (5) propose a nearest-neighbor procedure that could be used when studying the variation of modularity in geographic space. The approaches outlined here, readily extendable to non-morphometric datasets, allow study of the variation in the degree of integration between *a priori* defined modules. A Java application – that will allow performance of the proposed test using a software with graphical user interface – has also been developed and is available at the Morphometrics at Stony Brook Web page (http://life.bio.sunysb.edu/morph/).

## Introduction

Organisms, to function as a whole, need their parts to be connected and establish relationships, i.e. they need a degree of “integration” [1]. However, the integration between parts is not uniformly distributed but there are units – called “modules” – that are relatively independent from each other. Modularity is of interest for evolutionary biologists as an increase of the level of modularity – i.e. an increase in the level of independence between modules – is believed to increase evolvability as modular organization allows the modules to evolve independently [2]–[4]. Modular structure can be recognized at multiple levels of biological organization and, in the case of variational modularity in morphology (i.e. modular organization that can be inferred from the higher degree of covariation within modules relative to the level of covariation between modules; [5]), it is assumed to reflect evolutionary or developmental processes that result in modularity itself [6]. For instance, traditionally two modules are recognized in the house mouse mandible (i.e. the alveolar region and the ascending ramus). These regions reflect the presence of multiple morphogenetic units and provide evidence of some degree of genetic modularity due to pleiotropic effect [7]–[13]. Analyses of morphological modularity and integration are extremely popular nowadays and many methods for studying patterns of modularity and integration exist [14]–[15]. Klingenberg [16] has proposed the use of the Escoufier RV coefficient [17] as a measure of the level of modularity in geometric morphometric datasets. The RV coefficient is a measure of the covariation between blocks of variables relative to the covariation within blocks, so this coefficient is an ideal choice as a measure of variational modularity (i.e. variation between modules relative to variation within modules). The Escoufier RV coefficient can be considered a multivariate extension of the expression for the squared correlation coefficient between two variables [16], ranging from 0 to 1 with lower values indicating lower covariation between modules relative to the variation within modules (i.e. higher degree of modularity). Robert and colleagues [18] investigated several statistical properties of the coefficient at sample sizes comprised between 100 and 1000, showing that it has small bias and small variance (and, therefore, high precision) when used as estimator, for finite samples, of the population levels of association between matrices. Klingenberg [16] also proposed using the RV coefficient to assess if the level of modularity of an *a priori* defined partition of anatomical landmarks in modules is higher than random partitions of the same set of landmarks. The method developed by Klingenberg [16] has been widely used since its development, being also implemented in the software package MorphoJ [19]. In fact, the ease with which estimates of the degree of modularity can be obtained and the hypothesis of organization in *a priori* defined modules (groups of landmarks) can be tested has promoted the use of such method on biological datasets. However, while comparisons of the levels of overall integration across groups of observations can be performed using measures of the dispersion of the eigenvalues of the principal components [14], no specific method to analyze variation in the RV coefficient between *a priori* defined groups of observations exists. This represents an interesting area of research as, if modularity promotes evolvability, then inferring variation in levels of modularity might highlight variation in the levels of evolvability. In a similar fashion, variation in levels of modularity might point out a variation in the processes that are responsible of modular organization. Reflecting the high potential interest of variation in the degree of modularity, studies have started reporting [20]–[21] the RV coefficients for different *a priori* defined groups of observations (different species in the case of the two cited studies). Jojić and colleagues [22], when comparing traditional and geometric morphometric approaches to the study of modularity, while cautioning against potential discrepancies between methods, suggested that direct comparisons among studies on the mouse mandible are reliable. However, it has been shown [23], using random data, that the RV coefficient decreases when sample size increases. If this was true for real morphometric datasets, then comparisons of RV coefficients across samples or studies with different sample sizes might be meaningless. Another shortcoming of simply reporting the RV coefficient is that, even in the case of equal sample sizes, it does not represent a formal test of the null hypothesis of no variation in levels of modularity but, at best, an exploratory approach.

Here, we show, using a real morphometric dataset, that the RV coefficient is, indeed, dependent on sample size. We then describe how a permutational approach can be used to test for the difference in RV coefficient between two *a priori* defined groups of observations, providing simulations of type I error for this new test. We, further, suggest two other possible ways of studying variation in modularity. In particular, how a rarefaction procedure could be used to obtain “sample-size-corrected” RV values and how a nearest neighbor procedure might be used to explore patterns of variation in the RV coefficient in geographic space.

## Materials and Methods

### Datasets Used

**Figure 1:**
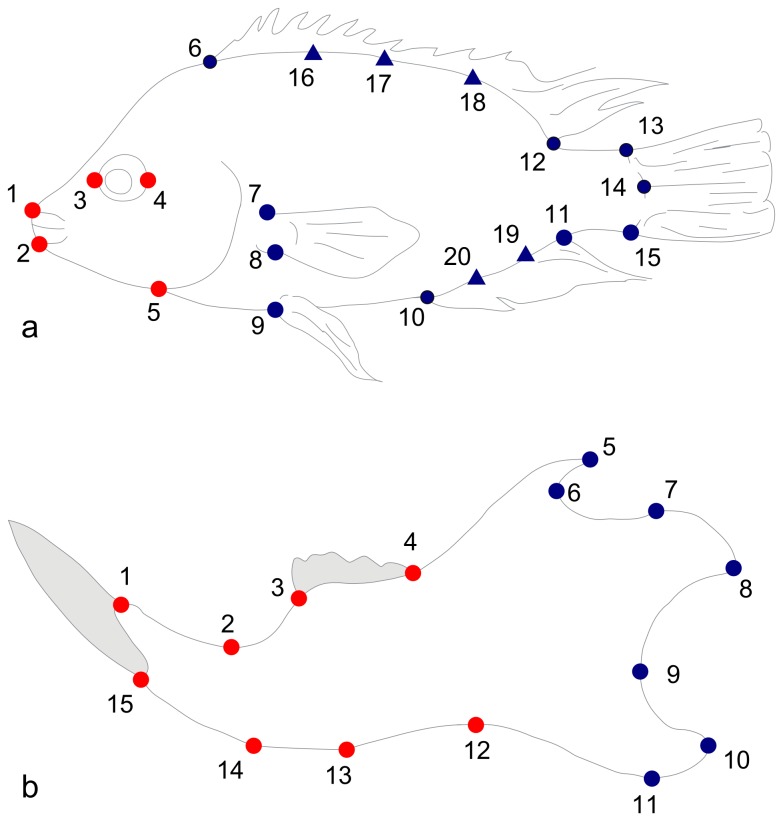
Configurations of points for datasets used in the present work. a. *Dataset1*, b. *Dataset2* and *Dataset3*. Circles represent landmarks, triangles semilandmarks. Red and blue distinguish the two modules.

In this study, three real datasets (Tab. 1, Fig. 1) were used either directly or to generate simulated data. They are unpublished datasets that will be used here just for the methodological purposes of this study while the analyses reflecting their biological relevance will be published elsewhere (P. Franchini, C. Fruciano et *al. unpublished data*). For all datasets, (semi)landmarks were digitized using tpsDig2 [24]. Landmark and semilandmark configurations were then aligned [25]–[26] in tpsRelw [27]. In all cases, the allometric component of shape variation was removed by performing, in MorphoJ [19], a multivariate regression of shape on centroid size. Residual Procrustes coordinates were then partitioned in two modules for the analyses of modularity (Tab. 1, Fig. 1). When performing Klingenberg’s method [16], significant modularity was found for all the datasets and subsets in Tab. 1 except for parental species and F1s in *Dataset1* and for the CD subset of *Dataset2*, probably as a consequence of the small sample size of these subsets. All the subsequent analyses and simulations were performed in MATLAB (MathWorks, inc.).

**Table 1:** Datasets used in the present paper.

Dataset		Subsets	
Description	n	Description	n
*Dataset1* Body shape data for a QTL experiment in Midas cichlid fish (P. Franchini, C. Fruciano et *al. unpublished* *data*). A total of 20 points, comprising both landmarks and semilandmarks, was digitized. The full dataset oflandmark/semilandmark configurations was then subjected to a generalized Procrustes analysis (GPA)with sliding of semilandmarks [26]. Two modules (one cranial and one post-cranial; [31]) were defined,thereby effectively partitioning the 40 variables into two groups of 10 and 30 variables respectively.	376	*Amphilophus astorquii*	16
		*Amphilophus zaliosus*	41
		*F1* individuals obtainedcrossing a female *A. astorquii*and a male *A. zaliosus*	11
		*F2* individuals obtainedbreeding two F1 individuals	308
*Dataset2* Data from a morphometric analysis of a contact area between two chromosomal races (CD and ACR)of the Western European house mouse (Franchini *et al*. *unpublished data*). Fifteen landmarks were collected inthe left mandible of each individual and then subjected to a GPA [25]. Two modules, defined by the ascendingramus and the alveolar region [9], were defined by 7 and 8 landmarks respectively, partitioning the variablesinto two groups of 14 and 16 variables.	84	CD	18
		ACR	66
*Dataset3* Data from a morphometric analysis of a hybridization area between two chromosomal races(CD and Standard races) of the Western European house mouse (Franchini *et al*. *unpublished data*).Landmarks and modules as in *Dataset2*.	86	Standard	45
		Hybrids	41

### Relationship between RV and Sample Size in a Real Dataset

To test if there is a relationship between the RV coefficient and sample size in real datasets, we performed a rarefaction procedure on the 308 configurations of Procrustes residuals belonging to F2 generation individuals in *Dataset1*. Briefly, we carried out the following procedure:: 1. we randomly sampled with replacement from the 308 configurations 100 samples for each of the sample sizes comprised between 10 and 300; 2. for each sample we computed the RV coefficient; 3. we calculated then the mean and standard deviation of the RV coefficient for each set of 100 samples at each sample size. We repeated the procedure described above 200 times and obtained grand means of the RV coefficients and average standard deviation by averaging the results of the 200 independent rarefaction analyses. We also computed the variance of the mean RV estimate across the 200 independent analyses. To assess the impact of different alignment procedures, we performed the above mentioned analysis both aligning all the 308 observations prior to the rarefaction procedure with a single generalized Procrustes analysis and performing separate generalized Procrustes analyses for each of the two modules. Further, to assess the effect of superimposition at each step, we carried out a simplified version of the procedure described above (steps 1–3). In this simplified procedure we performed a generalized Procrustes analysis (both performing a single alignment for all the landmarks and using separate alignments for each module) for each of the samples obtained at step 1 of the procedure for all the sample sizes comprised between 10 and 300 at steps of 5 (10, 15, 20,…).

### Using Rarefaction to Standardize RV to a Given Sample Size

Here we suggest that using a rarefaction procedure could be useful to overcome the problem of dependency of Escoufier RV on sample size when one needs to compare RV values directly or use them in downstream analyses. We apply this idea to the main groups (*A. astorquii*, *A. zaliosus*, F1 specimens, F2 specimens) of *Dataset1*. In particular, we draw – sampling with replacement –1000 random samples of 11 observations (sample size of the smallest group, F1 individuals) from each of the groups, compute the Escoufier RV coefficient for each dataset and, finally, compute a mean RV coefficient for each group.

### Permutation Test of the Null Hypothesis of no Difference in RV Coefficient

Here we propose to test for the difference in RV between two *a priori* defined groups of observations.

Let A and B be two matrices *n x t* and *m x t* of, respectively, *n* and *m* observations and *t* variables (constituting, effectively, two *a priori* defined groups of *n* and *m* observations). Let *t = q*+*r* where *q* and *r* are two *a priori* defined groups of variables (representing modules). We propose computing the difference between RV coefficients for each group, defined as:
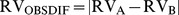



Where RV_A_ and RV_B_ are, respectively, the RV coefficient between q and r variables for matrix A and the RV coefficient between the same two sets of variables for matrix B.

We propose – to test the null hypothesis 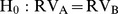 – creating two new groups A_PERM_ (of size *n* x *t*) and B_PERM_ (of size *m* x *t*) resampling without replacement observations from the pooled sample M of size (*n*+*m*) x *t* and computing the difference in RV between the two new groups as:
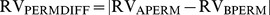



Where RV_APERM_ and RV_BPERM_ are the RV coefficients computed on A_PERM_ and B_PERM_, respectively. After repeating the procedure N_PERM_ times to obtain an empirical distribution of RV_PERMDIFF_, we will consider the proportion of times in which RV_OBSDIFF_ exceeds the differences of the empirical distribution as the probability level at which the null hypothesis H_0_ of no difference in RV (modularity) between the two groups can be rejected.

We used this permutation test on *Dataset2*, comparing the RV coefficients for mice from the CD and ACR groups, and on *Dataset3* comparing mice with standard and hybrid karyotype (in all cases 1000 random permutations were used).

### Analysis of Type I Error for the New Permutation Test

To analyze the type I error for the permutation test proposed here, we used two different approaches: simulating no difference in modularity using real datasets and simulating no difference in modularity using random data.

For the first approach, we independently used the following sets of landmark configurations: F2 individuals from *Dataset1*, CD mice mandibles from *Dataset2* and ACR mice mandibles from *Dataset2*. For each of the sets of landmark configurations independently and for each of the sample sizes comprised between 40 and 200 at steps of five, we generated 1000 random multivariate normal datasets with the observed mean and covariance. Then we subdivided each dataset in two subsets of equal number of observations (rounded to the next integer) and performed the proposed permutation test for the difference in RV coefficient. We, finally, used the proportion of significant (at the 5% probability level) tests at each sample size as a measure of type I error.

For the second approach, to test type I error in different conditions (different number of total cases, different number of total variables, different number of variables for each block, different number of observation for each *a priori* defined group), we generated 200,000 random datasets. We used random datasets as this choice allowed us to have a wide range in the number of variables and their possible subdivision in two blocks. Each dataset had a random number of observations comprised between 40 and 200 and a random number of variables comprised between 20 and 100. Observations for each variable were drawn from the standard uniform distribution on the open interval (0,1). Each random dataset was, further, divided into two groups of observations and two blocks of variables with the number of observations per group being random but constrained to be higher than 20 and the number of variables per block random but constrained to be at least four. For each of the simulated datasets we performed the proposed permutation test for the difference in the RV coefficient, using the proportion of significant tests as a measure of type I error. To investigate if the type I error of our test was dependent on number of cases, number of variables, difference in sample size between groups, difference in variables between blocks, we also produced plots of such features for datasets which returned a significant test.

### Using a Nearest Neighbor Approach to Analyze Variation in Modularity in Geographic Space

In the case of a relatively small number of discrete samples with multiple observations (sampling sites), variation in geographic space could be studied by standardizing to a common sample size through rarefaction and/or multiple pairwise comparisons using the permutation test proposed here. However, in case of sparse sampling in geographic space, we suggest that a solution could be to compute, for each *x_i_* observation in geographic space, the RV coefficient for the subset comprising *x_i_* itself and its *k-*nearest neighbors, computing also the average spatial coordinates of the subset. In this way, for each of the original observations an RV coefficient and a new spatial position will be computed. The new matrix representing the variation in the RV coefficient in geographic space could then be analyzed with exploratory or hypothesis-testing tools. For instance, plots of the variation of the RV coefficient in geographic space can be obtained and functions to describe the observed patterns can be fitted to the data. Here we provide an example of such an approach using *Dataset1*. We divided a hypothetical bidimensional square – representing a hypothetical geographic space – into four quadrants. Then for three of the four quadrants we generated – using the multivariate normal distribution having the mean and covariance matrix of the F2 individuals in *Dataset1*–100 observations per quadrant and assigned them random uniform coordinates within the quadrant. For the fourth quadrant we generated 100 observations from the multivariate normal distribution having the mean and covariance matrix of a subset of F2 individuals chosen so to have a higher RV coefficient and assigned them random uniform coordinates. In such a way, we simulated the situation where within one quadrant – the upper left – there would be a maximum in the RV coefficient. We then computed the RV coefficient with the *k*-nearest neighbor approach outlined above using *k* = 20, Euclidean distances in the two-dimensional space as distance measure and the KD-tree technique for finding the *k*-nearest neighbors [28]. Finally, we fitted a third-order polynomial on the obtained matrix of RV coefficients and average spatial coordinates and we plotted the fitted surface as a surface plot and as a heat map.

## Results

### Relationship between RV and Sample Size in a Real Dataset

**Figure 2:**
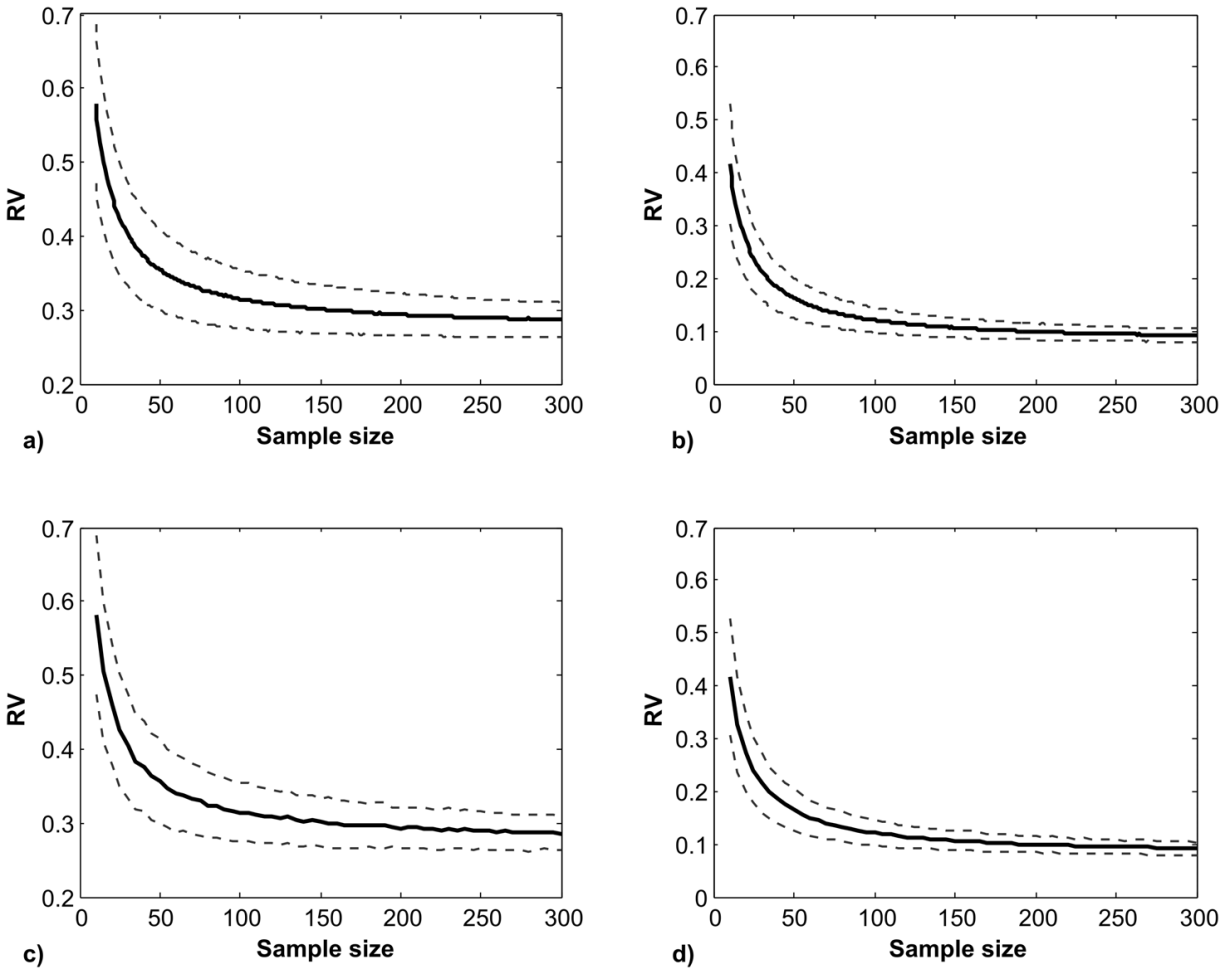
RV at different sample sizes obtained from the rarefaction analysis. Solid line: mean, dashed lines: mean +/− standard deviation. a. Single generalized Procrustes analysis on the complete dataset. b. Separate Procrustes superimpositions for each module on the complete dataset. c. Generalized Procrustes analysis on each randomly drawn sample, using the full configuration of landmarks. d. Generalized Procrustes analyses on each randomly drawn sample, performing separate superimpositions for each module.

Fig. 2 shows the average RV coefficient at each sample size obtained through rarefaction analysis. A clear pattern of decrease in RV at increasing sample sizes can be observed. Such pattern is especially pronounced at sample sizes smaller than 100, thus suggesting that comparing the RV coefficient across samples with different sizes can be misleading. It is interesting to notice that lower overall values and lower variances of estimates of the RV coefficient are obtained when performing separate generalized Procrustes analyses for each module (Fig. 2a,b). On the other hand, performing generalized Procrustes analyses for each dataset obtained resampling from the population does not affect the general finding of a decrease in the RV coefficient at increasing sample sizes (Fig. 2). The variance of the estimated average RV coefficient, as expected, decreases at increasing sample sizes (Fig. S1) and, while a critical sample size cannot be identified, the decrease is very pronounced at lower sample sizes.

### Using Rarefaction to Standardize RV to a Given Sample Size

In our example dataset, the average RV obtained after a rarefaction analysis at the smallest sample size (n = 11) for *A. astorquii* was 0.704, for *A. zaliosus* 0.623, for the F1 specimens 0.668, for the F2 specimens 0.554. These results might suggest that modularity in *A. astorquii* x *A. zaliosus* hybrids behaves as a transgressive trait. However, here the dataset has been just used as an example of using rarefaction to standardize the RV coefficient and any biological consideration will be made elsewhere.

### Application of the Permutation Test to Real Datasets

The RV coefficients of mice belonging to the groups CD and ACR from *Dataset2* were, respectively, 0.31 and 0.20 (difference 0.11; sample sizes were, respectively, 18 and 66). However, the newly developed permutation procedure did not reject (p = 0.758, based on 1000 permutations) the null hypothesis of equal RV coefficients. On the other hand, using the same test on standard and hybrid mice from *Dataset3* (RV coefficient 0.31 and 0.20, respectively, with sample sizes 45 and 41) resulted in significant differences (p = 0.02). The fact that in the two datasets, the same difference in the RV coefficient produced non-significant and significant results, respectively, suggests that the observed difference in RV between the two groups in *Dataset2* can be explained as a consequence of their difference in sample size.

### Analysis of Type I Error

**Figure 3:**
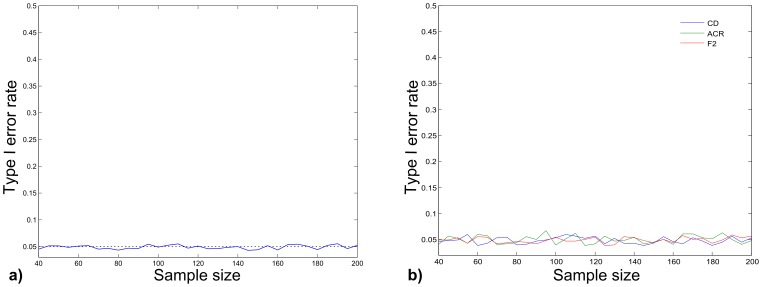
Type I error rate at different sample sizes. a. Average type I error rate across the full set of simulations using mean and covariance matrices of real datasets. b. Type I error rate for each of the simulations based on real datasets.

In the analysis of type I error using mean and covariance from real datasets, the average type I error for all simulation was 0.049. Plots of type I error at different sample sizes (Fig. 3) do not show any relationship between type I error rate and sample size.

In the analysis of type I error using random datasets with variable number of cases, number of observations, number of observations in each group and number of variables in each block, the overall type I error was 0.05. Plotting the number of cases, number of observations, fraction of observations in the first group, fractions of variables in the first block, difference between number of variables and number of cases for occurrences of type I error showed no departure from random expectations (Figure S2). This shows that type I error for the suggested permutation test is not influenced by the analyzed features.

### Using a Nearest Neighbor Approach to Analyze Variation in Modularity in Geographic Space

**Figure 4:**
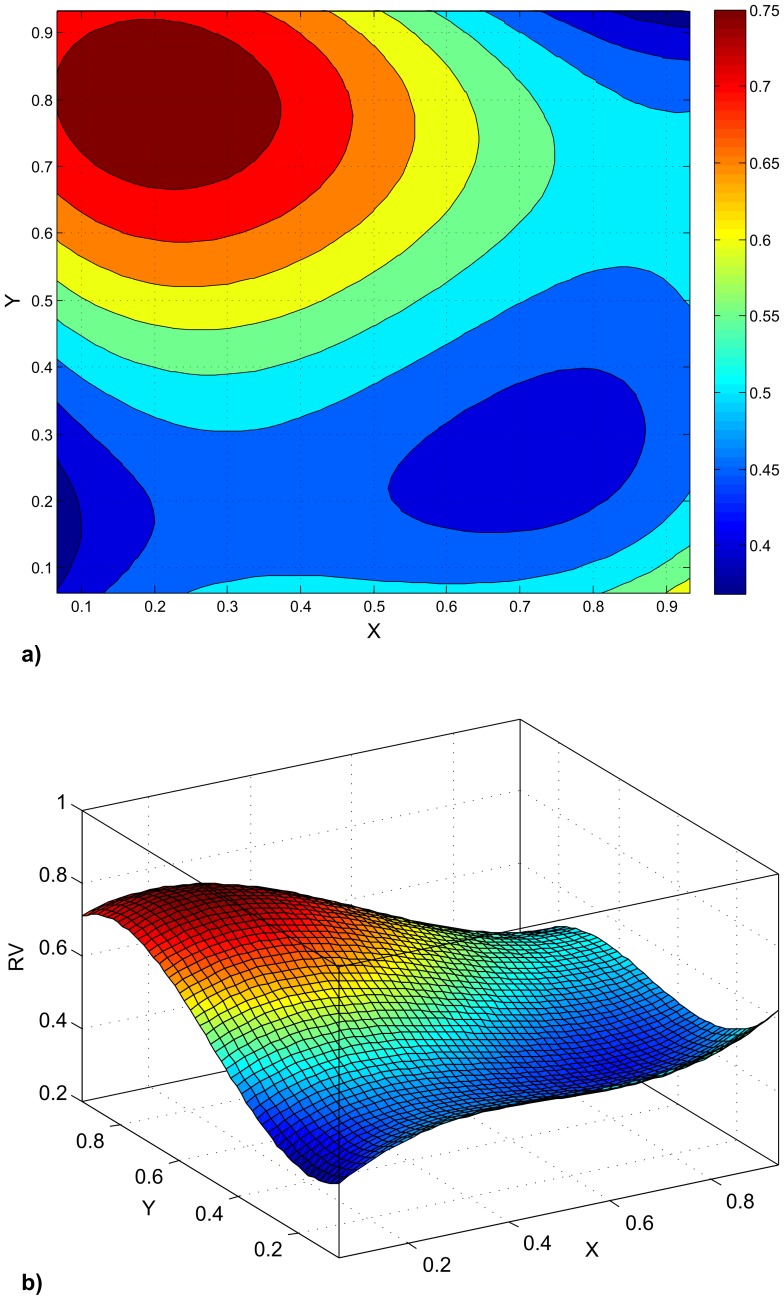
Variation of the RV coefficient in geographic space. Computation follows the *k*-nearest neighbor approach outlined in the paper and the predicted values at each site were then modeled using a third-order polynomial. a. Heat map. X and Y represent coordinates in a bi-dimensional space, the color reflects the value of RV predicted by the polynomial fitting. b. Surface plot. The basis represents the two dimensional space, the elevation the predicted RV. Color coding as in a.

In our example analysis using randomly distributed observations, a third-order polynomial provided a reasonably good fit (R^2^ = 0.69) for modeling the variation of the RV coefficient in a bi-dimensional space. A surface plot and a heat map are provided in Fig. 4. Looking at the plots, a maximum in the RV coefficients can be noticed in the upper left quadrant, which is the one we chose to contain the maximum.

## Discussion

In the present paper we have dealt with the problem of studying variation in morphological modularity. We have shown that the value of the Escoufier RV coefficient, despite being a useful and appealing measure of the strength of integration between modules, is dependent on sample size, even in real morphometric datasets. In particular, our results show that at sample sizes smaller than 100, the relationship between the RV coefficient and sample size is non-linear. Especially at sample sizes lower than 50, the RV coefficient rapidly decreases at increasing sample sizes. It is relevant to notice that sample sizes lower than 50 or 100 per group are quite common in modularity studies and the dependence of the RV coefficient on sample size makes comparisons of modularity between groups with different number of observations unreliable. In addition to this, mere comparisons of the RV coefficient values do not test the null hypothesis of no difference between groups of observations. Here we presented a permutation procedure to address the limitations outlined above by testing the null hypothesis of no difference between two groups. We have shown that such a procedure has appropriate type I error and that type I error is not dependent on factors such as total sample size, difference in sample size between groups, total number of variables, difference in number of variables between modules, difference between number of variables and number of cases.

Studies of morphological modularity are becoming increasingly popular, also because of the development of easy to use software tools to investigate the existence of multiple modules within a configuration of landmarks. Some studies have also started to report the RV coefficient for multiple species [20]–[21]. In this paper we also suggested that a rarefaction procedure could be used to derive “sample size-standardized” values of the RV coefficient to be used in downstream analyses. Our suggestion can have multiple applications. For instance, RV values derived from such a rarefaction procedure could be used in phylogenetic comparative studies to investigate the variation in the level of modularity across phylogenies [29]–[30]. Finally, we also suggested the use of a *k*-nearest-neighbor procedure to create groups of individuals of equal sample sizes in space computing then the RV coefficient on each group. Our example, involving sparse observations in a two-dimensional space, reflects one obvious possible application of such a nearest-neighbor procedure: the study of variation in modularity across geographic space. However, given that *k*-nearest-neighbors can be computed on spaces with number of dimensions higher than two, this idea could be used to explore different problems. It should be noticed that this method will always find one or more *maxima* and *minima* even using random data. Therefore, using a polynomial fit on the results of the *k*-nearest-neighbor approach could represent a useful exploratory approach while hypothesis-testing approaches should be used in case one wants to test for significance of the variation in geographic space. We consider our ideas of using rarefaction to obtain sample size-standardized RV values and using a *k*-nearest-neighbor approach to study RV variation in space as mere suggestions as the statistical properties of both approaches should be carefully investigated under a variety of conditions and we consider such investigations beyond the scope of the present paper. However, our results showing high variance of the rarefaction estimates at small sample sizes suggest caution when using resampling to obtain sample-size corrected RV values to be used in downstream analyses. At the same time, the sudden drop in the variance of mean RV estimates observed at increasing sample sizes suggests that even moderate increases in sample sizes could result in a sensibly better estimation of a sample-size corrected RV value. On the other hand, preliminary simulations of the *k*-nearest-neighbor method (data not shown) assuming one *maximum* in one quadrant at different values of *k* (10–25) show that the average distance between absolute *maxima* across different values of *k* is around 10% of the maximum distance between observations, thus suggesting that the *maximum* is found in the same region, even at different *k*.

We believe that the methods presented here represent new tools for the study of variation in morphological modularity, and precisely in the analysis of how the level of integration between *a priori* defined modules co-varies with other categorical or continuous variables. We also developed a Java application with graphical user interface to perform in a user-friendly way the proposed permutation test for the difference in modularity between *a priori* defined groups. While we explicitly developed and tested these methods on landmark-based geometric morphometric datasets, we believe that they could be easily applied, perhaps with minor modifications, to non-morphological studies of modularity. For instance, if modular groups of co-expressed genes are identified in gene expression studies, it would be straightforward to study the variation in modularity among different samples.

## Supporting Information

Figure S1 **Variance across 200 independent simulations of the average RV coefficient obtained through rarefaction at different sample sizes using a single generalized Procrustes analysis (GPA) for all landmarks or separate GPAs for the two modules (** **Fig. 1a** **).**
(PDF)

Figure S2 **Frequency of different features in the cases of type I error.** Blue: observed frequencies, red: expected frequencies simulating random data using the conditions under which the type I error simulations have been run (between 40 and 200 observations, between 20 and 100 variables, first group of at least 20 observations, first module of at least 4 variables).
(PDF)
