# Supplementary figures and images for: Resampling-Based Approaches to Study Variation in Morphological Modularity
Source: PLoS One. 2013 Jul 16;8(7):e69376. doi: 10.1371/journal.pone.0069376 (PMC3712944; doi:10.1371/journal.pone.0069376)

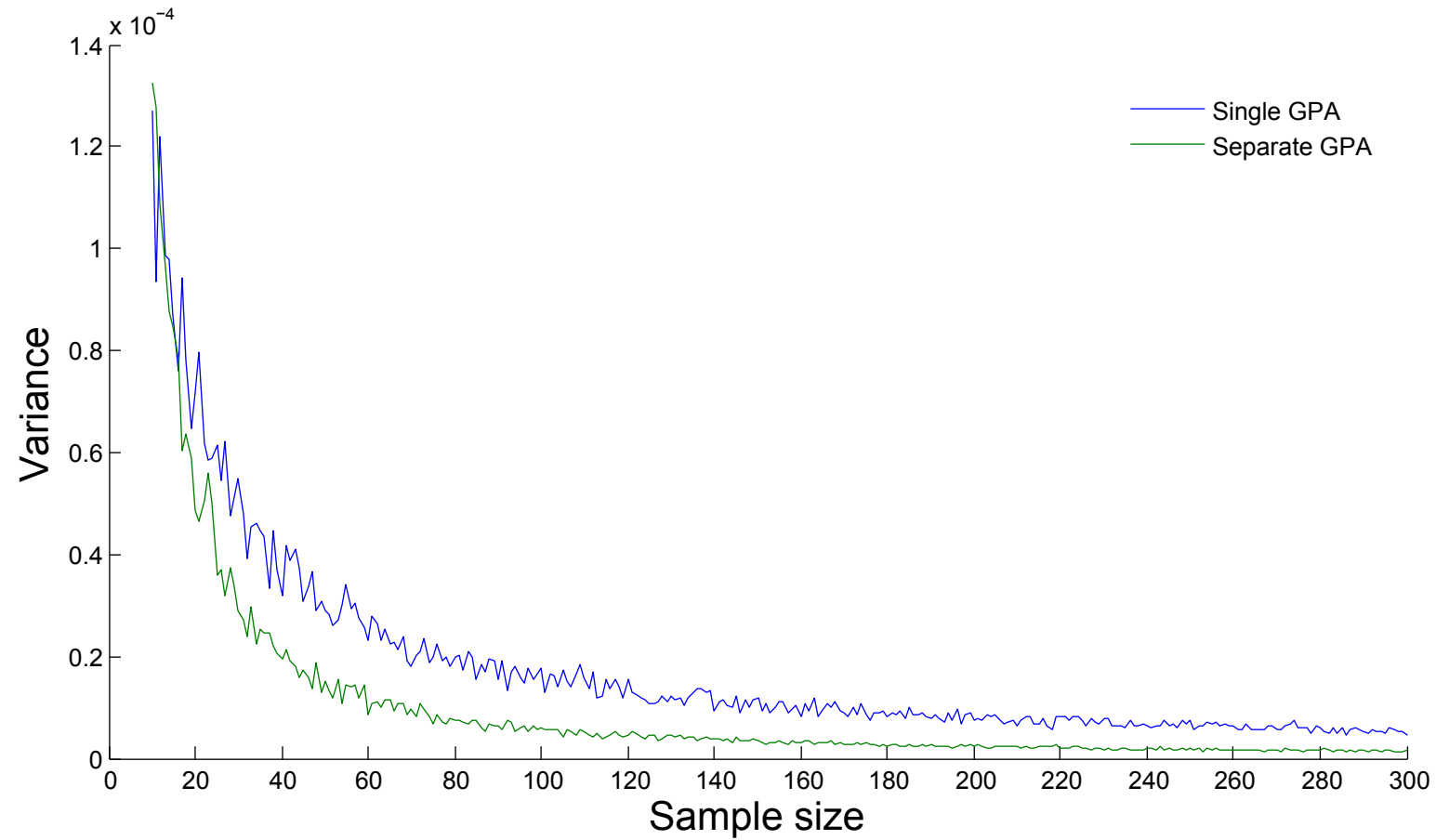

Supplement: Figure S1 — Variance across 200 independent simulations of the average RV coefficient obtained through rarefaction at different sample sizes using a single generalized Procrustes analysis (GPA) for all landmarks or separate GPAs for the two modules ( Fig. 1a ). (PDF) [file pone.0069376.s001.pdf]

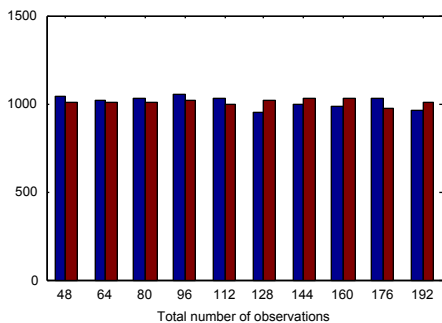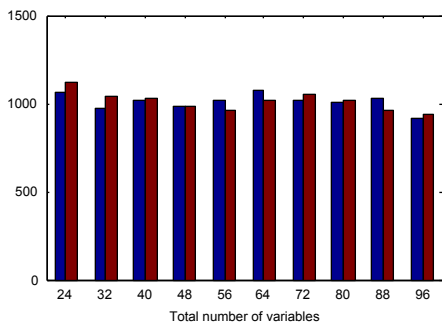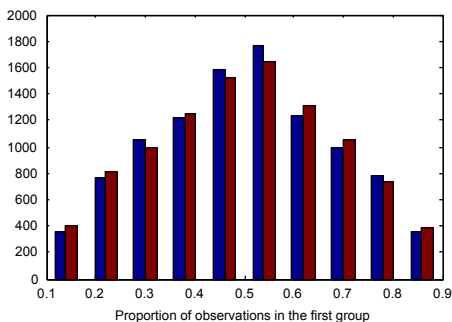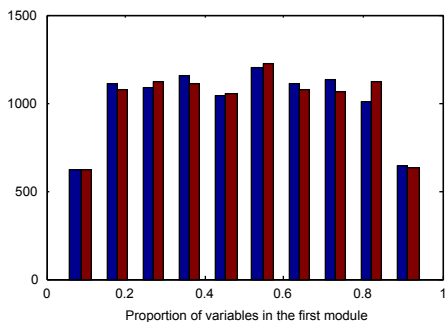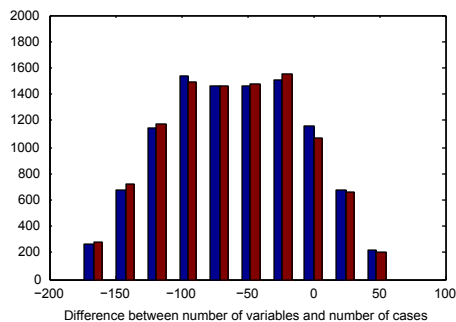

Supplement: Figure S2 — Frequency of different features in the cases of type I error. Blue: observed frequencies, red: expected frequencies simulating random data using the conditions under which the type I error simulations have been run (between 40 and 200 observations, between 20 and 100 variables, first group of at least 20 observations, first module of at least 4 variables). (PDF) [file pone.0069376.s002.pdf]
